# Determination of pholcodine alone or in combination with ephedrine in human plasma using fluorescence spectroscopy

**DOI:** 10.1038/s41598-022-13194-1

**Published:** 2022-06-07

**Authors:** Heba Elmansi, Fathalla Belal, Galal Magdy

**Affiliations:** 1 Pharmaceutical Analytical Chemistry Department, Faculty of Pharmacy, Mansoura University, P.O. Box 35516, Mansoura, Egypt; 2 Pharmaceutical Analytical Chemistry Department, Faculty of Pharmacy, Kafrelsheikh University, P.O. Box 33511, Kafrelsheikh, Egypt

**Keywords:** Chemistry, Analytical chemistry

## Abstract

In this study, sensitive, facile, and cost-effective spectrofluorimetric approaches were developed for the determination of pholcodine and ephedrine. Method I is a novel spectrofluorimetric method depending on measuring the native fluorescence of pholcodine at 337 nm after excitation at 284 nm over a concentration range of 0.01–2.4 μg/mL. The method sensitivity reached quantitation and detection limits down to 10.0 and 5.0 ng/mL, respectively. Method II relied on the simultaneous estimation of pholcodine and ephedrine using synchronous fluorimetry for the first time. The cited drugs were measured concurrently at 286 and 304 nm for pholcodine and ephedrine, respectively at Δλ of 40 nm without interference. Excellent linear relationship between concentration and response was obtained over the ranges of 0.05–6.0 μg/mL and 0.02–1.0 μg/mL for pholcodine and ephedrine, respectively. The method showed distinct sensitivity and exhibited quantitation limits of 20.0 and 10.0 ng/mL and detection limits of 10.0 and 5.0 ng/mL, respectively. The method was successfully applied to the syrup dosage form. The two developed approaches were also applied to in-vitro plasma samples, showing good bioanalytical applicability and providing further insights for monitoring drug abuse. The proposed methods were validated according to ICHQ2(R1) guidelines. The proposed methodologies' greenness profiles were evaluated using two greenness assessment tools.

## Introduction

It is critical to ensure the safety and efficacy of pharmaceutical dosage forms through quality control guidelines. When one or more of the active substances in the dosage form has the potential to be abused, the significance of this fact is amplified^1^. Opiate alkaloids are naturally occurring pain relievers that are most well-known for their capacity to create euphoria and addiction risk. Semisynthetic opiates are used as anti-tussives, analgesics, or sedatives^2^.

**Figure 1:**
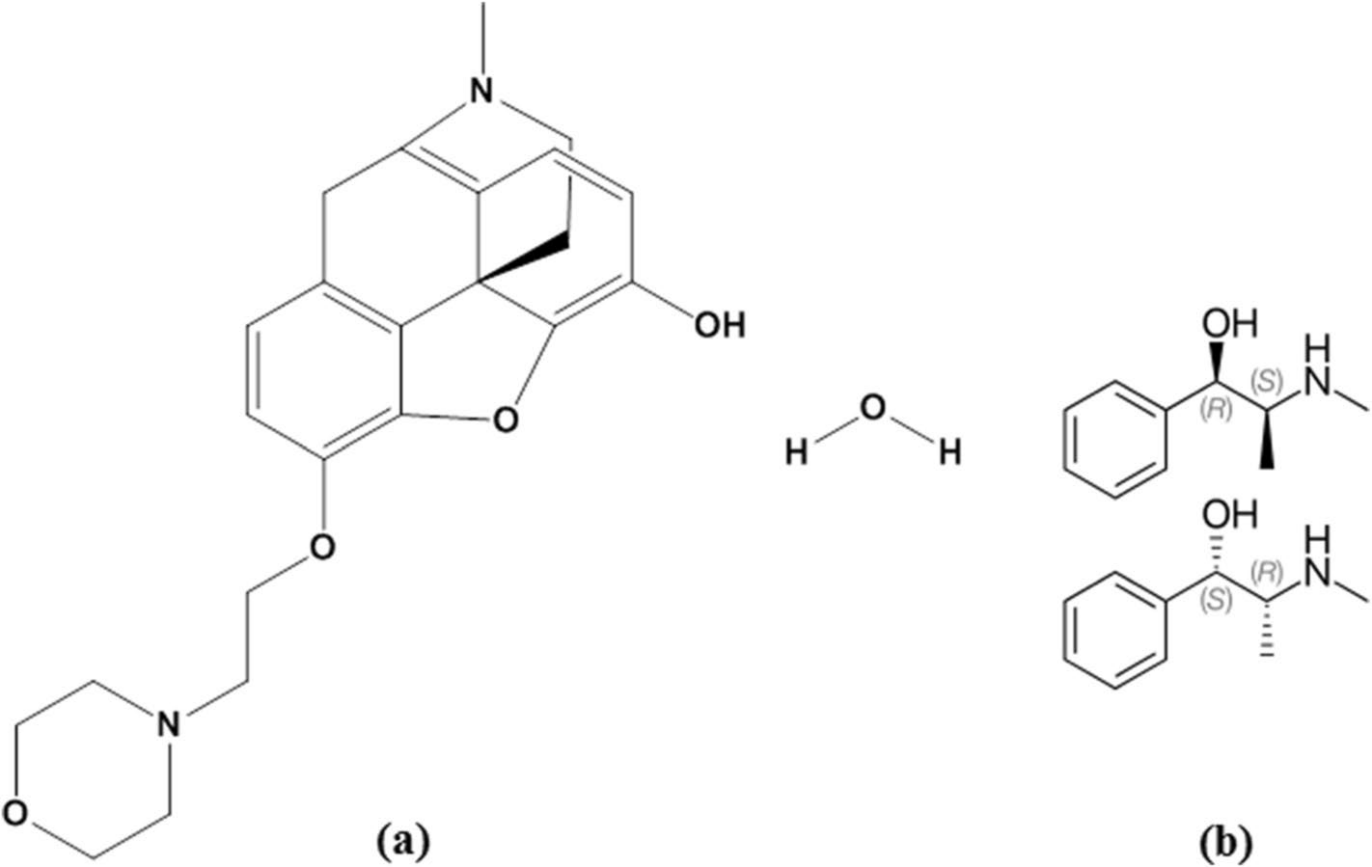
Chemical structure of the studied drugs: (**a**) PHL (**b**) EPH.

Pholcodine (PHL) ((4R,4aR,7S,7aR,12bS)-3-methyl-9-(2-morpholin-4-ylethoxy)-2,4,4a,7,7a,13-hexahydro-1H-4,12-methanobenzofuro[3,2-e]isoquinolin-7-ol;hydrate, Fig. 1a)^3^, a semi-synthetic derivative of morphine, is classified as a cough suppressant acting centrally with similar effects and usage to dextromethorphan. It can treat unproductive coughs and shows a moderate sedative effect^4^. In the United States, PHL is classified as a Schedule I drug. In Egypt, PHL is classified as a substance subject to drug restrictions according to Table 3 attached to the Egyptian Drug Law No. 182 of 1960^5^. The European Medicines Agency (EMA) has recently updated its comprehensive benefit/risk analysis^6^. As a result, there is an urgent need to develop a sensitive and rapid analytical approach for PHL analysis in order to manage its potential abuse.

Ephedrine (EPH) ((1R,2S)-2-(methylamino)-1-phenylpropan-1-ol, Fig. 1b)^7^, a sympathomimetic amine, is usually prescribed as bronchodilator and decongestant. It reduces swelling, constricts blood vessels in the nose, and widens the lung airways, making breathing easier^4^.

The current trend in cough treatment is to use medications with two or more active ingredients that work in synergistic manner to provide the best clinical benefit as decongestant, antitussive, and antihistaminic^4^. The combination of PHL and EPH is indicated for the relief of upper respiratory symptoms and nonproductive cough caused by common cold^8^.

Variable methods for determination of PHL have been reported including UV/Vis spectrophotometry^9^, spectrofluorimetry^10^, high-performance liquid chromatography (HPLC)^11–14^, gas chromatography (GC)^15^, chemiluminescence^16^, non-aqueous titration^17^, and electrochemistry^5,18^. Similarly, EPH was determined using spectrophotometric methods^19–22^, HPLC^23–25^, capillary electrophoresis^26–29^, GC^30,31^, LC-MS^30,32,33^, and electrochemistry^34,35^. While the methods reported for the simultaneous quantitation of PHL and EPH mixture were spectrophotometric^9^, HPLC^8,36^, and TLC methods^8^.

To date, no spectrofluorimetric approaches were reported for the concurrent analysis of such mixture. In addition, no native spectrofluorimetric methods for PHL determination have been reported, only one derivatization method based on a condensation reaction was reported^10^. Accordingly, the main objective of this work was to develop a highly sensitive native spectrofluorimetic method for PHL analysis (Method I) and to develop a synchronous spectrofluorimetric method for resolving and estimating the mixture of PHL and EPH for the first time (Method II) with no need for pre-derivatization or complicated steps.

When compared to other conventional analytical techniques such as UV–visible spectrophotometry, electrochemical methods, and chromatographic methods, the spectrofluorimetric technique is a well-known analytical tool that aims to increase the method's simplicity, selectivity, and sensitivity without affecting precision^37,38^. It is also available in most laboratories and provides a cost-effective analytical solution without the need for sophisticated instruments as in HPLC, LC–MS, and GC^39^. In addition, synchronous spectrofluorimetry is of great importance in the analysis of mixtures having overlapped spectra. The advantages of synchronous spectrofluorimetry include high sensitivity, increased spectral resolution, greater selectivity, less light scattering, and spectral simplicity^40^. As a result, synchronous spectrofluorimetry can be utilized as an effective analytical technique for quantitative determination of several pharmaceuticals in a single run due to its narrow, sharp spectrum^41^.

The two methods were efficiently employed for estimation of PHL and EPH in spiked human plasma samples with acceptable percent recoveries due to their high sensitivity and selectivity. The importance of these methods is significantly increased when one of the analyzed drugs is abused. The two methods are rapid, cost-effective, and economic. Furthermore, the proposed approaches' greenness profiles were considered using two greenness evaluation tools; Analytical greenness metric approach (AGREE) in addition to Green Analytical Procedure Index (GAPI), and they were confirmed to be green methods^42,43^.

## Experimental

### Materials and reagents

PHL monohydrate and EPH samples were kindly provided by Amoun Pharmaceutical Co. (El-Obour City, Cairo, Egypt). Human plasma samples were obtained from Mansoura University Hospitals (Mansoura, Egypt) and were kept frozen at − 80 °C until usage. Organic solvents were purchased from Tedia, High Purity Solvents, Fairfield OH, USA. Different surfactants, boric acid, borax, sodium hydroxide, acetic acid, sodium acetate, phosphoric acid, and sodium dihydrogen phosphate were obtained from El-Nasr Pharmaceutical Chemicals Company (ADWIC), Cairo, Egypt.

Surfactant solutions were prepared in a concentration of (1.0% w/v or v/v) using distilled water. 0.2 M of acetate, borate, and phosphate buffers were freshly prepared according to USP. Throughout the study, double distilled water and spectroscopic grade solvents were employed whenever needed.

### Instrumentation

Cary Eclipse Fluorescence Spectrophotometer with Xenon flash lamp from Agilent Technologies (Santa Clara, CA 95,051, United States) was used for fluorescence measurements. It was operated at high voltage mode (800 V) at 5 nm slit width. The medium voltage mode (600 V) was used for biological study. Synchronous spectrofluorimetric readings were recorded at Δλ of 40 nm. Vortex mixer, IVM-300p (Gemmy industrial Corp, Taiwan), pH-meter, Jenway 3510 (UK), centrifuge, 2-16P (Germany), and syringe filters with pore size of 0.45 µm (Phenomenex, USA) were employed in the biological study.

### Preparation of stock solutions

PHL and EPH standard solutions (100.0 μg/mL) were obtained by dissolving 10.0 mg of the drug in methanol in a 100 mL volumetric flask. Subsequent dilutions using the same solvent were followed to get the working solutions. The produced solutions remained stable for 7 days at 4 °C.

### Procedures

#### Calibration curves

##### Method I

Working solutions with final concentrations ranging from 0.01 to 2.4 μg/mL were prepared by transferring aliquots of PHL standard solution into a set of 10-mL volumetric flasks and completing to the mark with acetonitrile. After excitation at 284 nm, the corrected fluorescence intensities (FI) were measured at 337 nm. Blank samples were tested similarly. The calibration curve and hence the regression equation were constructed by graphing the corrected fluorescence intensities versus the respective drug concentrations in μg/mL.

##### Method II

Aliquots of standard solutions were transferred into a set of volumetric flasks (10 mL) and completed to the mark with methanol to reach final concentrations in the range of 0.05-6.0 and 0.02-1.0 μg/mL for PHL and EPH, respectively. At Δλ of 40 nm, the intensities of synchronous fluorescence (SF) spectra for PHL and EPH were measured at 286 nm and 304 nm, respectively with blank samples measured similarly. Calibration graphs and the corresponding regression equations were computed by plotting the corrected SF intensities against the final drug concentration.

#### Determination of PHL/EPH synthetic mixtures

Aliquots of PHL and EPH working standard solutions were transferred into volumetric flasks (10 mL) to prepare three synthetic mixtures with different ratios within the range of each drug. The procedure detailed in “Method II” section was then followed. The corrected SF intensities were measured, and the corresponding regression equations were used to calculate the concentration of each drug.

#### Analysis of PHL and EPH in dosage forms

Because of the un-availability of the commercial preparation in the local market, prepared syrup containing both PHL and EPH was formulated by adding: 4 mg PHL and 7 mg EPH with benzoic acid, methyl paraben, propylene glycol, saccharin sodium, citric acid anhydrous, glycerin, sucrose, alcohol 96%, and distilled water. Different volumes were transferred and diluted with methanol to reach the studied concentration ranges. Subsequently, the procedure for calibration curves' construction in Method II was followed.

#### Procedures for spiked human plasma

##### Method I

Into a series of centrifugation tubes (15-mL capacity), 1.0 mL aliquots of human plasma were transferred separately to each and spiked with known volumes of PHL stock solutions to prepare three samples within the linear range. The spiked samples were subjected to vortex mixing for 2 min. A dilution to 5 mL was made with acetonitrile for protein precipitation. The tubes were then centrifuged for 20 min at 3000 rpm. 1.0 mL aliquots of the supernatant from each tube were filtered through syringe filters (0.45 μm) and transferred into 10 mL volumetric flasks. The procedure described under “Method I” section was then followed in parallel with the blank experiment, and then the corresponding regression equation was derived.

##### Method II

Into a set of 15.0 mL centrifugation tubes, 1.0 mL aliquots of human plasma were transferred separately to each and spiked with known volumes of PHL and EPH stock solutions to prepare three synthetic mixtures. The same aforementioned steps were repeated. The concentration of each drug was computed as mentioned under “Method II” section in parallel with the blank experiment, and then the corresponding regression equations were derived.

## Results and discussion

### Spectral characteristics

**Figure 2:**
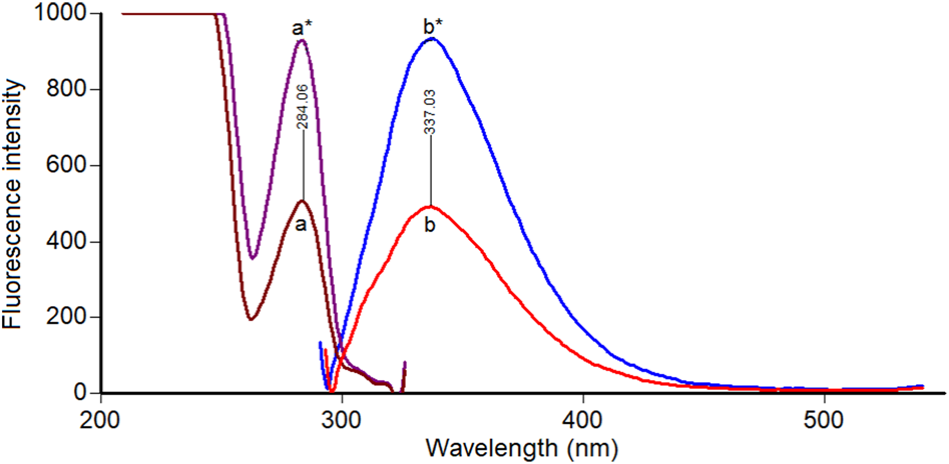
Excitation (a, a*) and emission (b, b*) spectra of PHL in acetonitrile at concentrations of 1.2 and 2.4 μg/mL.

PHL, being an isoquinolin-7-ol derivative, is expected to show high native fluorescence owing to the extended conjugation and benzene ring^44^. After excitation at 284 nm, PHL exhibited strong fluorescence at 337 (Fig. 2). This fluorescence was the base of a sensitive facile method for its determination (Method I).

**Figure 3:**
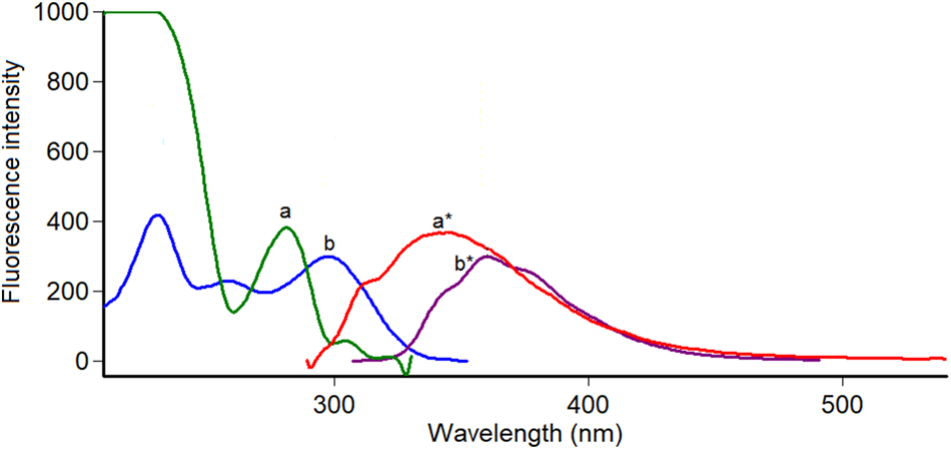
Fluorescence spectra of 1.2 μg/mL PHL (a, a*) and 0.2 μg/mL EPH (b, b*) in methanol.

**Figure 4:**
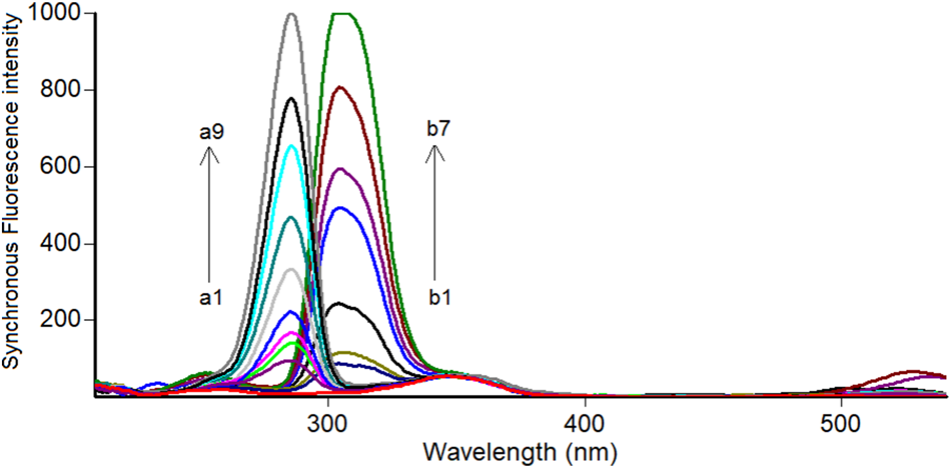
Synchronous fluorescence spectra of increasing concentrations of PHL (a1-a9: 0.05–6.0 μg/mL) and EPH (b1-b7: 0.02–1.0 μg/mL) in methanol at Δλ of 40 nm.

**Figure 5:**
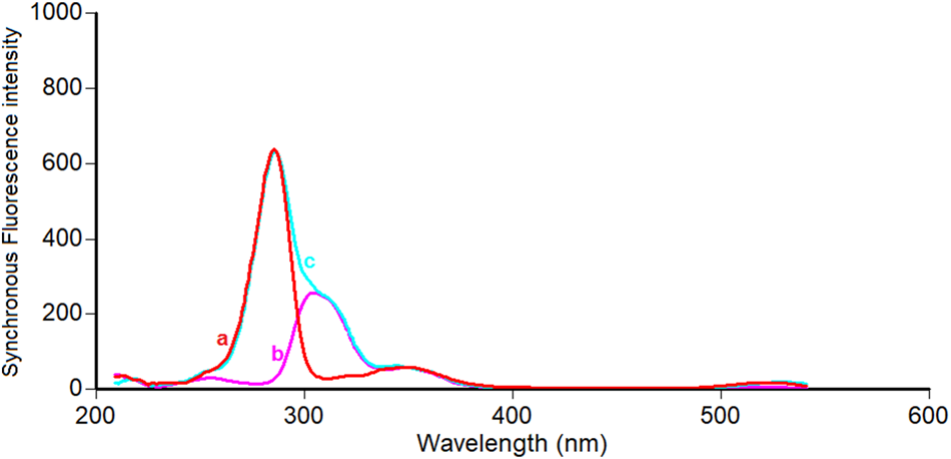
Synchronous fluorescence spectra of (a). PHL (4.0 μg/mL), (b). EPH (0.2 μg/mL), (c). synthetic mixture of PHL and EPH at Δλ = 40 nm.

Close inspection of Fig. 3 revealed that, the spectra of PHL and EPH are significantly overlapped which hinders their simultaneous analysis. Hence, method II was dedicated to resolving and estimating the mixture of PHL and EPH using highly selective and sensitive synchronous spectrofluorimetric technique. PHL and EPH synchronous fluorescence spectra were collected, and their peaks were found to be highly resolved which allowed each drug to be analyzed without interference from the other. Hence, the SF intensity of PHL and EPH could be measured at 286 nm and 304 nm, respectively (Fig. 4). All spectra were recorded at Δλ of 40 nm. A synthetic mixture of PHL and EPH proved the selective determination of each drug in presence of the other as illustrated in Fig. 5.

### Optimization of experimental conditions

The influence of various parameters on the fluorescence intensities of the two drugs was investigated and optimized.

#### Diluting solvent

**Figure 6:**
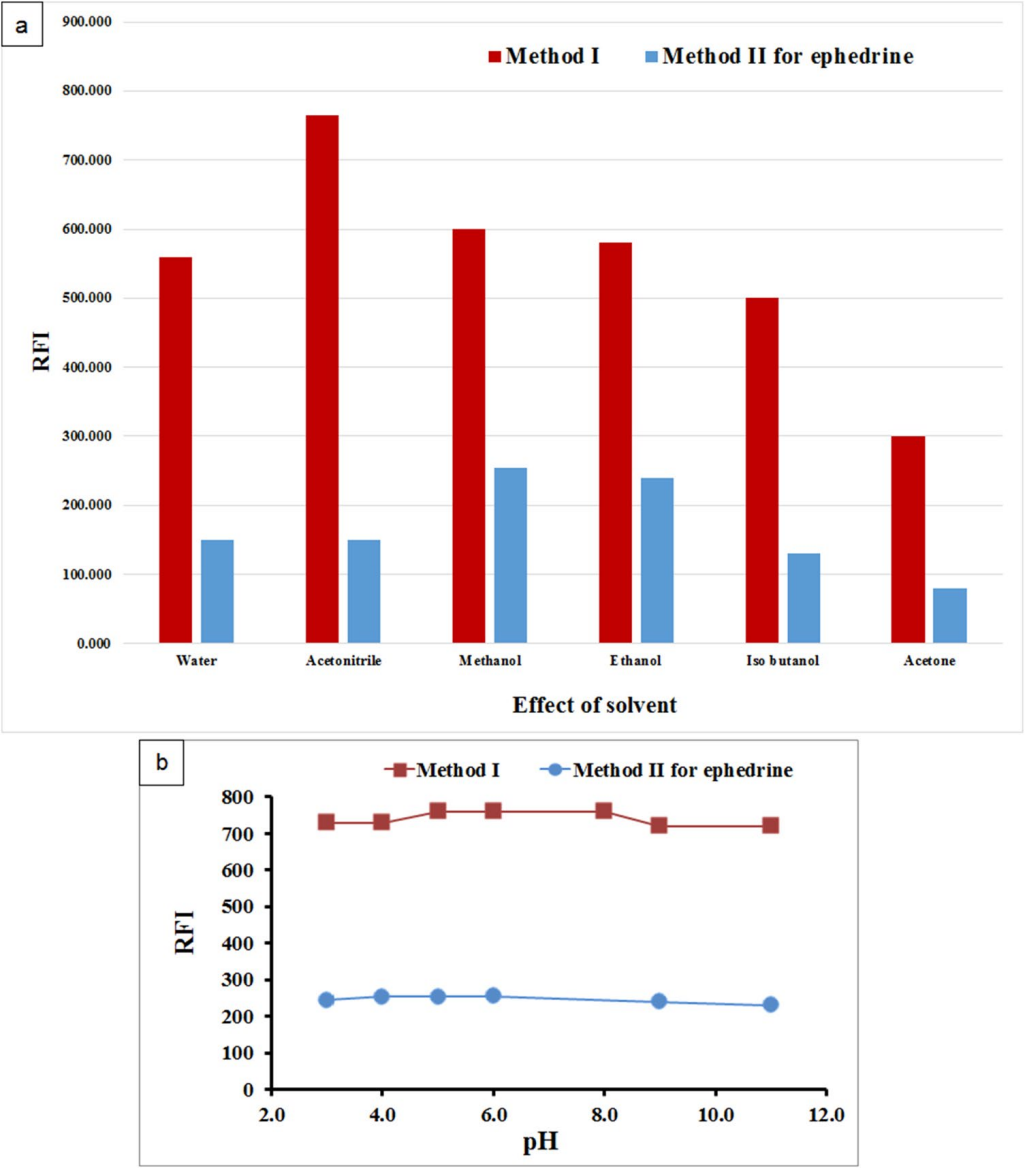
Effect of diluting solvent (**a**), pH (**b**) on synchronous fluorescence intensity of PHL (2.0 μg/mL) in method I and EPH (0.2 μg/mL) in method II.

Distilled water, methanol, acetonitrile, ethanol, iso-butanol, and acetone were all tested as diluting solvents. In method I, acetonitrile resulted in the highest SFI for PHL followed by methanol and ethanol. In method II, methanol resulted in the highest resolution, and therefore it was selected as the best diluting solvent for both PHL and EPH (Fig. 6a).

#### Effect of pH

Acetate, borate, and phosphate buffers were used to test the influence of pH over the whole pH range of 3–11. Acidic or basic pH values did not result in a significant improvement of fluorescence intensities in both methods (Fig. 6b). As a result, no adjustment of pH was required.

#### Study of organized media

Various organized media were evaluated for their potential capability to enhance the fluorescence characteristics of PHL and EPH by using concentrations greater than their critical micelles concentrations^45,46^. The investigated organized media included macromolecules such as carboxymethyl cellulose, and β-cyclodextrin, as well as surfactants such as tween-80, sodium dodecyl sulfate, and cetrimide. Because no substantial increase in fluorescence intensities was obtained, the study was conducted without the use of any organized media.

#### Optimization of Δλ

The value of Δλ has a significant impact on the synchronous fluorescence properties regarding sensitivity and resolution. It has a significant impact on the spectral pattern, signal's value, and synchronous band width^47,48^. PHL and EPH SF spectra were examined at a wide Δλ scale (20–140 nm). The optimal Δλ for PHL and EPH was found to be 40 nm, providing the best peak shape and well-resolved spectra. Decreasing or increasing Δλ adversely affects the fluorescence intensity as well as resolution for both drugs.

### Method validation

The proposed approaches were validated in consistence with the ICHQ2(R1) guidelines^49^.

#### Linearity and range

Method I showed a linear response by plotting the drug concentrations in μg/mL versus FI and method II showed linearity between drug concentrations in μg/mL against SFI. The acquired ranges, regression equations, and data of calibration curves are summarized in Table 1. The linearity of the calibration curves was validated by high values of correlation coefficients (r) and statistical analysis of the results^50^.

**Table 1:** Analytical performance data for pholcodine and ephedrine by the proposed spectrofluorimetric methods.

Parameter	Method I	Method II	
	PHL	PHL	EPH
λ_ex_- λ_em_	284–337 nm	Δλ = 40 at 286 nm	Δλ = 40 at 304 nm
Concentration range (μg/mL)	0.01–2.4	0.05–6.0	0.02–1.0
Slope	346.32	131.21	925.59
Intercept	70.59	89.40	67.09
Correlation coefficient (r)	0.9999	0.9997	0.9999
S.D. of residuals (S_y/x_)	3.00	8.22	7.34
S.D. of intercept (S_a_)	2.02	5.31	4.43
S.D. of slope (S_b_)	1.42	1.49	7.89
Percentage relative standard deviation, % RSD	1.07	1.66	1.50
Percentage relative error, % Error	0.43	0.62	0.57
Limit of detection, LOD (μg /mL)*	0.005	0.01	0.005
Limit of quantitation, LOQ (μg /mL)*	0.01	0.02	0.01

*Limits of quantitation and detection were experimentally evaluated.

#### Limits of quantitation (LOQ) and detection (LOD)

LOQ and LOD were experimentally evaluated by calculating signal to noise ratio (S/N) where: LOQ (S/N) ratio would be 10:1 and for LOD (S/N) ratio would be 3:1 (as per ICH guidelines Q2 R1). They are presented for the two methods in Table 1. The obtained results revealed that the proposed procedures can determine the investigated drugs with adequate sensitivity down to nanogarm levels and can be therefore utilized for their investigation in biological fluids.

#### Accuracy and precision

By comparing the obtained results with those provided by the comparison method^9^ (Supplementary Table S1), the accuracy of the two developed methods was investigated. The precision and accuracy of the two approaches were not significantly different, as shown in Table 2. It was proven by statistical assessment of the data via Variance ratio F-test and Student t-test, respectively^50^.

**Table 2:** Accuracy data for the proposed methods.

	Method I			Method II					
	PHL			PHL			EPH		
	Taken Conc. (μg/mL)	Found Conc. (μg/mL)	% recovery*	Taken Conc. (μg/mL)	Found Conc. (μg/mL)	% recovery*	Taken Conc. (μg/mL)	Found Conc. (μg/mL)	% recovery*
	0.01	0.0098	98.00	0.05	0.0503	100.60	0.02	0.0204	102.00
	0.2	0.2004	100.20	0.4	0.3932	98.30	0.04	0.0399	99.75
	1.0	0.9887	98.87	2.0	1.9479	97.40	0.2	0.1998	99.90
	1.2	1.2110	100.92	2.8	2.8243	100.87	0.4	0.4094	102.35
	2.0	2.0051	100.26	4.0	4.0436	101.09	0.6	0.5887	98.12
	2.4	2.3949	99.79	5.0	5.0800	101.60	0.8	0.7940	99.25
				6.0	5.9107	98.51	1.0	1.0079	100.79
Mean			99.67			99.77			100.31
± S.D			1.06			1.65			1.51
t**			0.04 (2.36)			1.05 (2.30)			0.54 (2.30)
F**			4.9 (19.29)			12.08 (19.32)			6.13 (19.32)

*Each result is average of 3 separate determinations.

**Values between parentheses are the tabulated values of t-test and F-test at *p* = 0.05^50^.

The proposed methods' inter-day and intra-day precisions were also investigated, with low percentage RSD and percentage error demonstrating acceptable precision of the proposed methods (Table 3).

**Table 3:** Precision data of pholcodine and ephedrine using the proposed spectrofluorimetric methods.

	Concentration(μg/mL)	Intra-day			Inter-day		
		${\overline{\text{x}}}$ ± S.D	% RSD	% Error	${\overline{\text{x}}}$ ± S.D	% RSD	% Error
Method I [PHL]	0.20	100.39 ± 1.82	1.05	0.61	100.75 ± 0.54	0.54	0.31
	0.60	101.14 ± 0.66	0.65	0.38	101.05 ± 0.87	0.86	0.50
	2.0	100.51 ± 0.91	0.90	0.52	100.65 ± 1.50	1.51	0.87
Method II [PHL]	0.4	100.83 ± 1.07	1.06	0.61	101.15 ± 2.53	2.53	1.46
	2.0	101.76 ± 0.55	0.54	0.31	100.58 ± 1.27	1.27	0.73
	4.0	99.81 ± 0.53	0.53	0.31	100.30 ± 1.86	1.86	0.76
Method II [EPH]	0.2	99.49 ± 1.98	1.99	1.15	98.94 ± 1.10	1.11	0.64
	0.6	100.67 ± 1.55	1.54	0.89	101.55 ± 0.97	0.95	0.55
	0.8	100.51 ± 0.91	0.90	0.52	98.50 ± 1.22	1.24	0.72

#### Selectivity

Method II was utilized for the concurrent estimation of PHL and EPH without interference from each other. Furthermore, the two approaches' selectivity was demonstrated by estimating the two drugs in complex matrices of plasma. It was proved that the methodologies have satisfactory selectivity to evaluate the studied drugs with acceptable percentage recoveries and low percentage RSD values, proving no interference from plasma components.

To confirm the method selectivity, the influence of other drugs that could be concurrently administered or formulated with PHL and EPH was also studied using Method II. The studied drugs included: carbinoxamine, chlorpheniramine maleate, and paracetamol. Interestingly; the proposed method could tolerate the mentioned drugs without affecting PHL and EPH fluorescence intensities as shown in Supplementary Table S2; indicating the high selectivity of the proposed method for determination of the studied drugs without interference.

### Applications

#### Analysis of PHL/EPH synthetic mixtures

Method II was used to analyze PHL and EPH in synthetic combination mixtures with varied ratios of the two drugs (Fig. 5). The drugs' concentrations in their synthetic mixture were estimated using the corresponding regression equation. The accuracy was ensured from the high values of percent recoveries as shown in Table 4.

**Table 4:** Analysis of laboratory prepared mixtures of pholcodine and ephedrine using synchronous spectrofluorimetric method (Method II).

	Proposed method			
	PHL		EPH	
	Amount taken (μg/mL)	% found*	Amount taken (μg/mL)	% found*
	4.0	100.86	0.05	101.47
	3.0	101.20	0.15	100.28
	0.10	100.34	0.10	99.69
Mean		100.80		100.48
± S.D		0.43		0.91
t**		0.46 (2.77)		0.76 (2.77)
F**		2.43 (19.00)		1.62 (19.00)

*Each result is average of 3 separate determinations.

**Values between parentheses are the tabulated values of t-test and F-test at *p* = 0.05^50^.

#### Analysis of PHL/EPH in their syrup

Method II was successfully employed for estimating the investigated drugs in their prepared formulated syrup. It is reported that PHL and EPH are formulated alone or with other drugs in a syrup formulation. The obtained results and those attained using a comparison method^9^ (Supplementary Table S1) did not differ significantly (Table 5). Statistical evaluation of the results adopting Student's t-test and Variance Ratio F-test^50^ indicated that the two approaches performed similarly regarding accuracy and precision, respectively.

**Table 5:** Determination of pholcodine and ephedrine in their prepared syrup by the synchronous spectrofluorimetric method (Method II).

Prepared syrup (4 mg PHL + 7 mg EPH)	Proposed method			
	PHL		EPH	
	Amount taken (μg/mL)	% Found*	Amount taken (μg/mL)	% Found*
	0.40	101.20	0.70	100.35
	0.10	100.95	0.175	100.43
	0.20	100.54	0.35	101.01
Mean		100.90		100.60
± S.D		0.33		0.36
t**		0.68 (2.77)		0.24 (2.77)
F**		3.25 (19.00)		2.46 (19.00)

*Each result is average of 3 separate determinations.

**Values between parentheses are the tabulated values of t-test and F-test at *p* = 0.05^50^.

#### Determination of PHL and EPH in human plasma samples

Assessment of PHL in biological samples is essential to evaluate the drug abuse issues^12^. Analysis of PHL in plasma was carried out following method I. Moreover, simultaneous estimation of PHL and EPH in spiked plasma was carried out regarding their therapeutic concentrations following method II. The maximum plasma concentration (C_max_) of PHL was found to be 12.8 µg/mL after administration of a 20-mg dose^51^, while C_max_ of EPH was stated to be 0.04–0.14 µg/mL (mean 0.08) when 22 mg/day dose was administered^52^. Method I reached PHL concentration down to 10 ng/mL. Method II sensitivity was down to 20.0 and 10.0 ng/mL for PHL and EPH, respectively. This high sensitivity permits analysis of the two drugs in plasma samples. Plotting the SFI or FI against the drug concentration in µg/mL in plasma samples spiked with PHL and EPH demonstrated a linear relationship (Table 6). High % recoveries (95- 107.5) were obtained, implying that the developed methods could be utilized to determine both drugs in spiked human plasma samples and confirming the validity of the method.

**Table 6:** Determination of pholcodine and ephedrine in spiked human plasma samples using the proposed methods.

	Method I		Method II		
	PHL		PHL	EPH	
	Amount taken (μg/mL)	% Found*	% Found*	Amount taken (μg/mL)	% Found*
	0.04	107.50	105.00	0.02	95.00
	0.50	99.00	99.40	0.05	102.00
	1.00	100.30	100.20	0.50	100.00
Regression equation	y = 88.563x + 31.204		y = 39.589x + 40.344	y = 221.93x + 2.4069	
Mean ± S.D		102.27 ± 4.58	101.53 ± 3.03		99.00 ± 3.61
Correlation coefficient (r)		0.9998	0.9999		0.9999

*Each result is average of 3 separate determinations.

### Evaluation of the greenness profile

**Figure 7:**
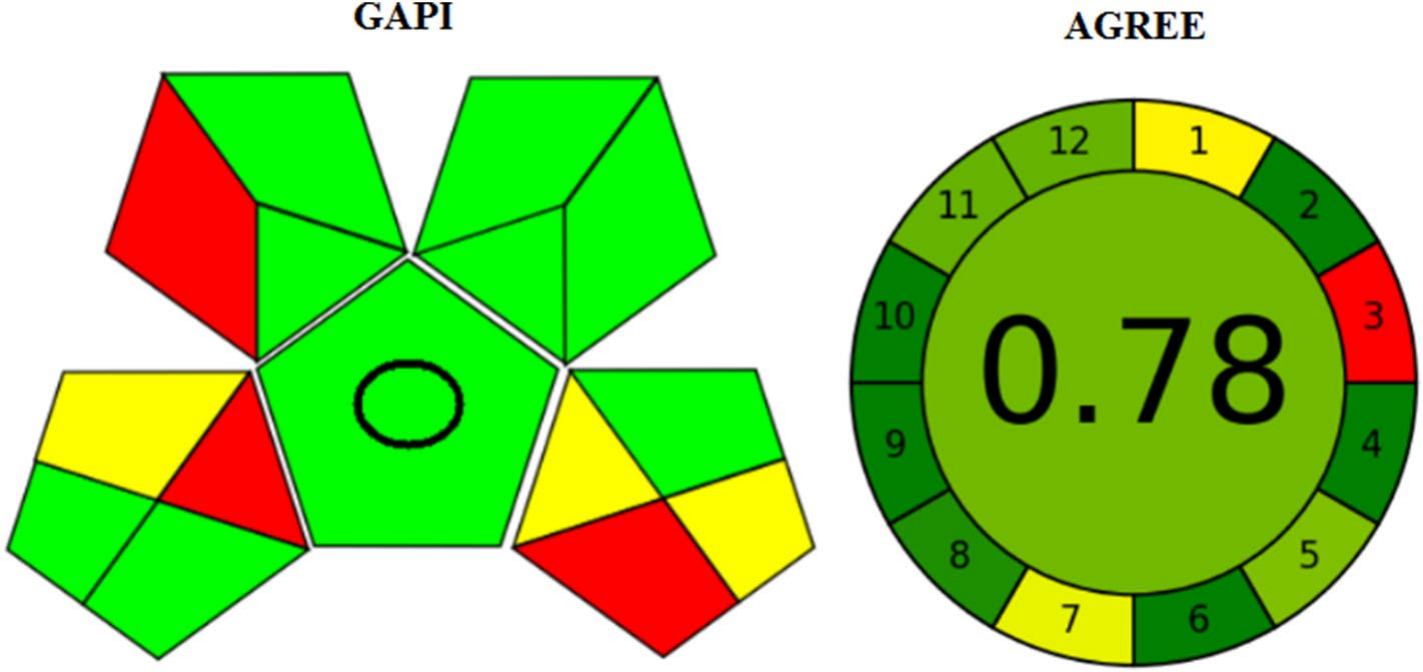
Results of greenness assessment of the proposed methods using AGREE and GAPI tools.

The importance of controlling waste and hazards to improve green analysis is increasing globally. In this study, we applied two green metric tools: Analytical GREENNESS metric approach (AGREE)^42^ and Green Assessment Profile Index (GAPI)^43^. Considering AGREE, it is a tool for determining the environmental and occupational hazards included in the analytical procedure by assessing 12 significant criteria. The final result is from 0 to 1.0. GAPI was also applied as a semiquantitative tool for determining the green property in each step. Both procedures need minimal amounts of non-toxic chemicals and produce minimal waste. Moreover, the designed methodologies are for qualification and quantification and they are also direct approaches. As shown in Fig. 7, the results are satisfactory indicating excellent green methodologies.

## Conclusion

This work is the first to present a synchronous spectrofluorimetic approach for estimating PHL and EPH simultaneously at Δλ of 40 nm without interference. A simple native spectrofluorimetric approach was also designed for the sensitive estimation of PHL with LOD of 0.005 µg/mL and over a concentration range of 0.01–2.4 µg/mL. The methods were successfully employed to assess the investigated drugs in spiked human plasma samples with high percent recoveries (95–107.5) owing to their high sensitivity. The suggested methods were successfully used to assess the two drugs in their syrup formulation. The developed methods offer numerous advantages, including being simple, rapid, cost-effective, and environmentally friendly. Furthermore, they provide excellent sensitivity, as well as wide linear ranges. The developed procedures were validated employing ICH recommendations, with accuracy, precision, and selectivity all falling within acceptable levels.

## Supplementary Information

Supplementary Information.

## Data Availability

The datasets generated and/or analyzed during the current study are available from the corresponding author on reasonable request.
