# Supplementary material for: Determination of pholcodine alone or in combination with ephedrine in human plasma using fluorescence spectroscopy
Source: Sci Rep. 2022 Jun 7;12:9372. doi: 10.1038/s41598-022-13194-1 (PMC9174196; doi:10.1038/s41598-022-13194-1)
Supplement: Supplementary file 1 — Supplementary Information. [file 41598_2022_13194_MOESM1_ESM.pdf]

Supplementary Information

Determination of pholcodine alone or in combination with ephedrine in human plasma using fluorescence spectroscopy

Heba Elmansi[1], Fathalla Belal[1], Galal Magdy[2,*]

[1] Pharmaceutical Analytical Chemistry Department, Faculty of Pharmacy, Mansoura University, Mansoura, P.O. Box 35516, Egypt

[2] Pharmaceutical Analytical Chemistry Department, Faculty of Pharmacy, Kafrelsheikh University, Kafrelsheikh, P.O. Box 33511, Egypt

***Corresponding author:** Galal Magdy.

E-mail address: galal_magdy@pharm.kfs.edu.eg

Supplementary Table S1: The data of analysis of pure samples, synthetic mixtures, and pharmaceutical preparations by the comparison method [9].

Comparison method [9]			
**Pure samples**			
**PHL**		**EPH**	
**Amount taken (µg/mL)**	**% found***	**Amount taken (µg/mL)**	**% found***
20.0	100.62	80.0	100.22
40.0	100.49	100.0	99.11
100.0	101.37	120.0	100.1
Mean	100.83		99.81
± S.D	0.48		0.61
**Synthetic mixtures**			
**PHL**		**EPH**	
**Amount taken (µg/mL)**	**% found***	**Amount taken (µg/mL)**	**% found***
20.0	99.99	80.0	101.42
40.0	101.32	100.0	102.12
100.0	100.45	120.0	99.86
Mean	100.59		101.13
± S.D	0.68		1.16
**Syrup**			
**PHL**		**EPH**	
**Amount taken (µg/mL)**	**% found***	**Amount taken (µg/mL)**	**% found***
20.0	100.31	80.0	100.72
40.0	100.25	100.0	101.24
100.0	101.32	120.0	100.11
Mean	100.63		100.69
± S.D	0.60		0.57

*Each result is average of 3 separate determinations.

Supplementary Table S2: Determination of PHL and EPH by method II in presence of commonly co-formulated drugs.

Interferent*	% Recovery		Standard deviation	
	PHL	EPH	PHL	EPH
**Carbinoxamine**	100.34	99.69	1.23	0.98
**Chlorpheniramine maleate**	100.21	99.46	1.51	1.50
**Paracetamol**	99.63	98.78	1.23	1.32

*Concentration of interfering drug is 1.0 μg/mL.
